# Household Hints and Recipes

**Published:** 1877-01

**Authors:** 


### Household Hints & Recipes.

CEMENT FOR VESSELS WITH VOLATILE FLUIDS.—According to Hirzel, a paste of finely powdered litharge and concentrated glycerine, makes a good cement, which hardens very readily.—*Med. Record.*

INK-STAINS may be removed from colored fabrics which would not bear the application of acids, chloride of lime, or other strong agents, by a concentrated solution sodium pyrophosphate; old stains do not yield at once, but require a prolonged application.

TO FASTEN IRON TO WOOD.—It is recommended to drop into the grove or hole bored in the wood a small quantity of sal-ammoniac dissolved in water. The effect of the salt is to cause the iron when placed in the wood to rust, and thereby to swell, making it difficult to part the two joined substances.

TO RESTORE COLOR.—When color on a fabric has been accidentally or otherwise destroyed by acid, ammonia is applied to neutralize the same, after which an application of chloroform will, in almost all cases, restore the original color. The application of ammonia is common, but that of chloroform is but little known.

PROGRESS OF HIPPOPHAGY.—In 1866 the first market for the sale of horse flesh was opened in Paris. In 1874 the Parisians consumed 3,659 horses, 496 asses and 29 mules, which yielded 1,295,520 kilos of meat, exclusive of the hearts, tongues, livers, etc. On the 1st of Jan. 1875, fifty markets for the sale of horse flesh existed in Paris.

MARKING INK OF TYRIAN PURPLE.—The linen or cotton goods to be marked are previously mordanted with a mixture of aluminum acetate and stannic chloride (tin crystals); after the spots have become perfectly dry, they are marked with a liquid prepared by saturating 5 grm. of ammonium carbonate with pure nitric acid and rubbing 3 to 4 grm. of carmine with the solution.

MUCILAGE FOR LABELS.—Those who are engaged in gumming labels are often annoyed by the curling up of the edges of corners. A remedy for this is to add to the mucilage a little salt, sugar, and glycerine, and the paper if not exposed to too strong a warmth, will not curl. A very small quantity of glycerine must be used, as a large proportion will prolong the process of drying.

TO PREVENT TROUBLESOME PERSPIRATION OF THE FEET.—Dr. I. H. Crawford, of Wiscassea (State not mentioned), writes to us that, in connection with the use of cold water and soap, the following formula has in his experience relieved this disagreeable affection:

Salicylic acid, gr. i.-ij.
Lycopodium powder, ℥i M

To be dusted on the feet.

A NEW ADHESIVE PLASTER.—A mixture of twenty parts of mucilage aud one part of glycerine constitutes an excellent shining and supple plaster, far cheaper than the resin and diachylon, and lasting more than a year without deterioration. Three or four layers of the mixture require to be spread over each other on the linen or other stuff, allowing sufficient intervals for the successive layers to dry.—*Journ. de Med. de Bruselles.*

INFANT FOOD.—Dr. Edward Smith, author of an excellent work on "Food," thinks that condensed milk is not a suitable food as a substitute for pure milk for infants. It is more fattening, but less nourishing, and greatly reduces the child's power of resisting diseases. Dr Smith states that children brought up on impure London-fed cows' milk will resist an attack of acute disease better than children fed on condensed milk.

PENCILS FOR COPYING.—Pencils are now made the marks of which may be copied in the same manner as writing made by the pen with ordinary copying ink. The method of preparing the lead is as follows: A thick paste is made of graphite, finely pulverized kaolin, and a very concentrated solution of aniline blue, soluble in water. The mixture is pressed into cylinders of suitable size, and dried, when it is ready for use. Gum arabic, it is said, may be substituted for the kaolin.

CLOSING CRACKS IN CAST IRON STOVES.—Good wood ashes are to be sifted through a fine sieve, to which is to be added the same quantity of clay finely pulverized, together with a little salt. The mixture is to be moistened with water enough to make a paste, and the crack of the stove filled with it. The cement does not peel off or break away, and assumes an extreme degree of hardness after being heated. The stove must be cool when the application is made. The same substance may be used in setting the plates of a stove, or in fitting stove pipes, serving to render all the joints perfectly tight.—*Scientific American.*

PREPARATION FOR WASHING BLUE.—The *Scientific American* gives the following receipt: Twenty lbs. white potato starch, twenty lbs. wheat starch, twenty lbs. Prussian blue, two lbs. indigo carmine, and two lbs. finely ground gum arabic are mixed in a trough, with a gradual addition of sufficient water to form a half fluid, homogeneous mass, which is then poured out on a board with strips tacked to the edges. It is then allowed to dry in a heated room until it does not run together again when cut. It is next cut by a suitable cutter, into little cubes, and allowed to dry perfectly. They are finished by being placed in a revolving drum, with a suitable quantity of dry and finely pulverized Paris blue, until they have a handsome appearance. The cost is about 12 cents per pound.

---

TOOTH POWDER.—The following is an excellent recipe:

White Castile Soap, powd., 4 ounces.
Orris-root, powd., 4 "
Cuttle-fish bone, powd., 6 "
Prepared chalk, powd., 6 "

Flavor with sassafrass or wintergreen, as desired. In the preparation of tooth-powder, great care should be taken to finely pulverize all the dry ingredients, and to reduce the hard and gritty ones to the state of impalpable powder. To ensure the perfect mixture of all the ingredients they should be stirred together until they form an apparently homogeneous powder, which should then be rubbed through a fine gauze sieve.—*Druggists' Advertiser.*

---

ENGLISH COURT PLASTER.—Take of isinglass, 10 parts. Dissolve it in a sufficient quantity of common hot water, so that a colature (solution) is obtained, which, should be 120 parts.

Take sixty parts of this solution, and, by means of a brush, spread a sufficient number of coatings on stretched silk (taffeta), which should measure, for each thirty grammes of isinglass, one hundred and four (104) centimetres in length and forty-two (42) centimetres in breadth.

Each coating should be allowed to dry.

The remaining sixty parts of this isinglass solution are gradually mixed with

Alcohol, 40.
Glycerine 1, and this mixture is brushed over the fabric in the same manner as the previous solution.

Finally, the reverse side of the fabric is coated with a sufficient quantity of tincture of benzoin, and the plaster is then well dried, and preserved in a dry place.

It is lustrous, and, when moistened, becomes very adhesive to the skin.

---

ELASTIC DAMMAR VARNISH.—The *Pharmaceutisches Centralhalle* gives the following process for making an elastic flexible varnish for paper, which may be applied without previously sizing the article:

Dammar in small pieces, 40 grains.
Acetone, 6 ounces.

Expose the whole to a moderate temperature for about two weeks, frequently shaking. At the end of this time, pour off the clear saturated solution of dammar in acetone, and add to every four parts of varnish three parts of rather dense collodion; the two solutions are mixed by agitation, the resulting liquid allowed to settle, and preserved in well closed phials. This varnish is applied by means of a soft beaver hair pencil in vertical lines. At the first application it will appear as if the surface of the paper was covered with a thin white skin. As soon, however, as the varnish has become dry, it presents a clear shining surface. It should be applied in two or three layers.

This varnish retains its gloss under all conditions of weather, and remains elastic; the latter quality adapts it especially to topographical crayon drawings and maps, as well as to photographs.

---

POISONOUS COOKING-UTENSILS.—The danger attending the use of porcelain-lined cooking-vessels, was pointed out at a meeting of the British Society of Public Analysis, by Mr. Robert R. Tatlock. He stated that the milk-white porcelain enamel with which cast-iron cooking-vessels are now so commonly coated, is in the highest degree objectionable, on account of the easy action on it of acid fruits, common salt, and other substances, by means of which lead and even arsenic are dissolved out in large quantity during the process of cooking. It was shown that it is not so much on account of the presence of large proportions of lead and arsenic that these enamels are dangerous, but because they are so highly basic in their character, and are so readily acted on by feebly-acid solutions. He thought that no enamel should be admitted to use unless it was totally unaffected by boiling with a one-per-cent. solution of citric acid, which was a very moderate test. Further, he gave it as his opinion that either the use of such poisonous ingredients as lead and arsenic in large quantity should be entirely discontinued, or that the composition otherwise should be of such a character as to insure that none of the poisonous substances could be dissolved out under ordinary circumstances.

